# Nanotechnology in oncology: a mini review

**DOI:** 10.1590/1806-9282.20241347

**Published:** 2024-12-02

**Authors:** Eyyup Cavdar, Kubilay Karaboyun, Yakup Iriagac

**Affiliations:** 1 Tekirdağ Namık Kemal University, Department of Medical Oncology – Tekirdağ, Turkey.; 2 Agri Ibrahim Cecen University, Training and Research Hospital, Department of Medical Oncology – Ağrı, Turkey.; 3 Balikesir Ataturk City Hospital, Department of Medical Oncology – Balıkesir, Turkey.

## INTRODUCTION

Improvements in cancer treatments are strengthening the ability of oncologists to tackle screening, diagnosis, and treatment. Increasing treatment successes in recent years have led to significant survival advantages even in advanced cancers, but cancer remains a global problem, accounting for approximately 10 million deaths in total and one in every six deaths per year^1^. Although substantial financial resources for cancer care, the lack of the desired cancer treatment outcomes and the increasing number of new patients with cancer have revealed the need for solutions other than the traditional methods for dealing with cancer.

Nanotechnology is considered to be a new power in healthcare, enabling the use of unique electrical, chemical, and magnetic effects with nanostructures obtained by using the quantum mechanics laws. Nanoscience and nanotechnology, which have the ability to provide big differences with small tools and instruments, an idea introduced by Nobel Laureate Richard P. Feynman, have made a rapid leap forward in the new century^2^. According to the National Nanotechnology Initiative, nanotechnology is the understanding and control of matter at sizes between about 1 and 100 nm, where unique phenomena enable new applications^3^. Reducing a normal-sized substance to nano-dimensions results in a change in its recognized characteristics, which means that nanotechnology cannot be expressed only in terms of size reduction. From macro-dimensions to nano-dimensions, a substance will transform optically, electrically, and chemically. A substance transforming from macro- to nano-scale transforms optically, electrically, and chemically, as well as having considerable magnetic power. Nanotechnology, which encompasses nanoscale science, engineering, and technology, includes the imaging, measuring, modeling, and processing of matter at this length scale. Because of all these properties, its use in medicine has also expanded widely and is particularly promising in oncology.

### Nanoparticles

Nanoparticles are small fragments <100 nm that contain unique properties in addition to the features of materials from which they originated. Being targetable and providing enhanced permeability and the ability to penetrate target tissues make them important in oncology. These capabilities of nanoparticles are powerful features for pharmacodynamics and bioavailability^4^.

**Figure 1:**
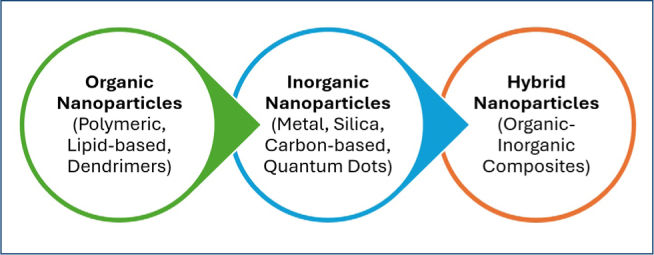
Nanoparticle types in oncology.

Nanoparticles utilized in oncology can be organic, inorganic, or hybrid (Figure 1).

### Imaging modalities

Traditional imaging modalities such as X-ray, computed tomography (CT), magnetic resonance imaging, and positron emission tomography–CT are the tools for screening and staging in current oncology practices. However, these imaging modalities may only be effective if the tumor exceeds a significant dimension or may not provide a definitive identification of benign lesions, leaving the final interpretation to the clinician.

In fact, nanoparticles have been developed currently for improved imaging detection. They can penetrate tumoral tissues much better and provide more specific information than conventional contrasts^5^. Metallic nanoparticles composed of heavy elements such as gold, platinum, silver, and cobalt enable fast penetration into tumoral tissues^6^ These heavy metals are important for the creation of the images. In addition, nanoparticles that are able to circulate in the bloodstream for a long time can proliferate in the tumor bed and enable the detection of smaller tumors. Recently, nanoparticles conjugated with tumor antibodies or tumor markers (such as carcinoembryonic antigen) are thought to be used in the initial phases of cancer formation and in the detection of early-stage cancer^7,8^. However, the hemophagocytic system detects these particles and prevents them from concentrating to the desired levels. Moreover, this can cause worse immunogenic challenges^9^. This is why the FDA requires blood compatibility tests before starting phase studies.

### Anticancer therapy

Nanoparticles may improve treatment efficacy, especially in cancer localizations where chemotherapy has poor efficacy, such as in the brain and liver. Current studies are promising^10,11^. In particular, their ability to increase the solubility and efficacy of conventional drugs while reducing their toxicity has led to studies.

Because of these properties, a large number of clinical trials and drug studies have taken place in nanomedicine. In 1995, the FDA approved doxil (liposomal doxorubicin), the first nanoparticle chemotherapy^12^. Just recently, daunorubicin, irinotecan, cytarabine, vincristine, paclitaxel, and leuprolide came into our lives as gifts of nanomedicine^13^. In recent years, it has also introduced new compounds to the benefit of oncologists.

Especially liposomal and protein nanoparticles are the most frequently used agents in chemotherapies. The possibility of having low toxic effects due to their organic structure may be one of the main factors for this. Moreover, they also have survival benefits. In a study by Gradishar et al., abraxan was found to be superior to conventional paclitaxel in terms of adverse events and also superior in terms of survival^14^. Besides using only passive targets, the addition of a ligand that can recognize a target to nanoparticles is promising for targeted therapies. However, there are more significant challenges to targeted therapies. The tumor cell needs to express adequate nanoparticle ligand-recognizing receptors. Specific ligand-recognizing receptors have to be unaffected by nanoparticles and sufficient concentrations of the drug separated from the particle in the intracellular environment are required for efficacy^15^. Currently, human epidermal growth factor-2 (HER2), transferrin receptor, folate receptor, and CD44 have emerged as targetable receptors. These receptors are being used for the management of breast cancer, hepatocellular carcinoma (HCC), malignant melanoma, lung cancer, and gastrointestinal malignancies^13^. In addition, while active targeted therapies prevent toxicity, they also improve treatment efficacy, as in the case of doxorubicin-containing anti-HER2 liposomes^16^.

Recently, T-cell therapy, vaccines, immunotherapies, and cytokine-mediated therapies have been the focus of nanotechnology. Nanoparticles can modulate T cells not only ex vivo but also in vivo. The CAR T-cell therapy is a particularly successful example of such a therapy. Nanoparticles targeting CAR T cells prolong the duration of treatment efficacy and prevent the development of resistance^17^. However, CAR T-cell therapy is expensive and few patients have access to it. On the contrary, immune checkpoint inhibitors, a powerful option for oncologists against cancer, are now increasingly being used. However, the tumor microenvironment in particular hinders the effectiveness of immunotherapy. In a study by Zhang et al., the combination of anti-PD1 iron oxide nanoparticles provided improvements in immunotherapy, while in a study by Kosmides et al., T cells could be dual activated by nanoparticles^18,19^. However, the immune system is not homogeneous, and recent research has revealed the existence of new subgroups, which is a barrier to nanoparticles^20^. Failure to produce targeted blockades or new immunosystem escape pathways may make it difficult to generate the desired immune response.

Radiotherapy in cancer treatment is an effective treatment option in patients with proper indications. However, dose modification is complicated, toxicity is higher, and there are treatment-resistant organ systems. Therefore, treatment planning is performed taking into account the tolerability of healthy tissue^21^. Nanomedicine aims to deliver radiotherapy more specifically to the tumor. In this way, it is possible to reach effective treatment doses while reducing toxicity^22^. Such purposes are achieved with nano-sensitizing particles. It also protects normal tissues by increasing radiosensitivity after penetration into the tumor. In addition, strengthening the collaboration between phototherapy and radiotherapy paves the way for new treatment modalities in oncology^23^. Table 1 briefly shows the relationship between radiotherapy and nanoparticles.

**Table 1:** Use of nanoparticles in radiation therapy.

Nanoparticle type	Function	Applications
Gold nanoparticles	Enhance radiation absorption	Tumor radiosensitization
Iron oxide nanoparticles	Magnetic guidance and hyperthermia	Imaging and tumor sensitization
Silica nanoparticles	Enhance targeting and drug delivery	Tumor-targeted drug release
Carbon-based nanoparticles	Radiation sensitization	Enhanced radiation treatment

In cancer patients, initial treatment leads to the death of most cancer cells, but those that cannot be destroyed develop new resistance mechanisms to survive and proliferate. In this situation, next-line treatment is initiated with drugs targeting new pathways, but the same condition may reoccur. Unfortunately, this recurrent phenomenon is one of the main problems that oncologists struggle to overcome. Nowadays, it is thought that resistance mechanisms, which may differ from patient to patient, can be overcome with individualized treatments. Depending on the tumor tissue taken from the patient, it is now possible with nanotechnology to manipulate or reproduce the target intracellular or extracellular molecule or receptor to overcome resistance^24^. Nanodrugs such as trastuzumab, pertuzumab, trastuzumab emtansin, lapatinib, neritinib, trastuzumab deruxtecan, and tucatinib for the HER receptor, which is the molecule that can be targeted from the resistance of chemotherapies used for years in HER2-positive breast cancers, one of the most well-known examples, strengthened the clinicians’ power. The production of a new drug after each resistance shows the progress of nanomedicine^25^.

### Future perspectives and current challenges

The use of nanotechnology in medicine is expanding, and the pressure for new discoveries is increasing. At the same time, however, the rapid rise has prompted debates about its potential risks and safety. The extent of the power of nanoparticles is not yet known and is recognized to be difficult to manage. Therefore, in clinical trials, medical researchers will have difficulties in securing informed consent from volunteers due to these uncertainties^26^. Nano-level interventions can deactivate some unknown control systems of cells and tissues, resulting in systemic effects. Research shows that nanoscale particles can be more damaging than larger particles^27^. It should also be noted that particles with high reactivity have catalytic effects, although their use in nanomedicine is less common.

While the FDA’s regulatory systems and regulations are often used to protect patient rights and privacy, other countries in Europe and Asia have also started to have a role in this area^28^. As artificial intelligence plays an active role and nanotechnology expands in parallel, the establishment of a global and centralized system may be the right legal and ethical way in the future. As medical researchers will be involved in these advancements, nanotechnology knowledge and education are gaining importance.

The high cost of nanotechnology is unlikely to be accessible to patients in developing or underdeveloped countries. New studies and new nanoparticles will enable this technology to be accessible to everyone.

## CONCLUSION

It is certain that the development of nanotechnology in medicine and the pharmaceutical industry will strengthen the position of cancer therapeutics. The introduction of targeted, more precise, and more effective therapies may be a solution to drug resistance while increasing the effectiveness of treatments. Moreover, it is strengthening our ability not only in treatment but also in imaging methods for cancer. However, for this important technology to be acceptable to all representatives of the health industry, new nanoproducts must undergo serious safety analyses. For sustainability, the entire process, from entry into the body to leaving the body or being eliminated, must be known. If the whole process is not known, it must be ensured that it is harmless. This technology, which is a major cancer-fighting power in our world, needs further studies and new discoveries.
